# Problematic internet use and its relationship with eating disorders

**DOI:** 10.3389/fpubh.2025.1464172

**Published:** 2025-01-23

**Authors:** Claudia Ruiz-Centeno, Raquel Cueto-Galán, Jose Miguel Pena-Andreu, Andrés Fontalba-Navas

**Affiliations:** ^1^Department of Public Health and Psychiatry, Faculty of Medicine, University of Malaga, Malaga, Spain; ^2^Biomedical Research Institute of Malaga, Malaga, Spain; ^3^Network of Artificial Intelligence Applied to Health (REDIAS), Malaga, Spain; ^4^Antequera Hospital, Northern Malaga Integrated Healthcare Area, Antequera, Spain

**Keywords:** problematic internet use, social media, eating disorders, anorexia, bulimia

## Abstract

Problematic Internet use has been associated with eating disorders. An increasing number of young adults are using social media, and a variety of content promoting anorexia and bulimia (pro-anorexia and pro-bulimia) has been identified. The aim of this study is to qualitatively analyze this content to understand how it affects individuals with eating disorders or those at risk. Content selection was carried out through non-random intentional sampling and based on the following criteria: the most prominent content on each platform in terms of the number of interactions, created within less than 5 years old, and displaying pro-ana and/or pro-mia content in English or Spanish. In total, 6 digital platforms were analyzed, including 57 resources (videos, images, blog entries, chat messages, PDF files, and group descriptions). The discourse was analyzed using both quantitative and qualitative approaches. The analyzed content includes numerous tips and tricks promoting eating disorders. Additionally, a widespread positive sentiment towards low weight and thinness was observed, while negative sentiments were associated with eating, gaining weight, etc. It has been demonstrated that self-esteem in adolescents and young adults can be affected by social media use, leading to body dissatisfaction that may result in increased use of these platforms with access to pro-anorexia and pro-bulimia content, which can contribute to the development of these disorders. The continuous variation and removal of this content, and the health problems it poses, requires further study of these digital resources and how users access them, in order to establish preventive measures to ensure health in the future.

## 1 Introduction

Technology is increasingly becoming part of the daily lives of adolescents and young adults, with numerous internet browsers and social media platforms (SM), where searching and finding content and information of all kinds is relatively easy (1–3). SM has become a community where users can communicate with each other and create, promote, or exchange diverse content (4, 5). The fact that internet access has become an integral part of the lives of children and adolescents is not without risks. Recent studies show that its use can negatively impact various areas of their lives, such as psychological wellbeing, relationhips with peers and family, academic performance, and daily functioning (6–8). This overuse of computers and smartphones has become a major public health issue, especially among adolescents (9). Young adults aged 18 to 25 have emerged as the predominant group among mobile Internet users, making up over a third of the total, with most being college students (10).

The latest results from the 2023 Social Media study, conducted annually by the Interactive Advertising Bureau (IAB) for digital advertising, indicate that 85% of internet users aged 12–74 use SM, which represents about 30 million individuals (11). Usage is slightly higher among women (87% vs. 83% men) and especially among those aged 18 to 24 years (94%). In 2023, users spent an average of 1:07 h on SM, compared to 55 min in 2019. The most used social networks are Whatsapp, Facebook, YouTube, Instagram, and Twitter. Users aged 12 to 34 are the ones who spend the most hours connected, especially on Spotify, Twitch, Discord, WhatsApp, TikTok, YouTube, Instagram, and Tinder. BeReal, TikTok, Reddit, and WhatsApp stand out as the networks that have increased their visit frequency the most. The network that has generated the highest volume of views this year was TikTok, with a growth of +109% compared to the previous year. Furthermore, the following of influencers has increased; Instagram remains the network where influencers are most followed, and there is a growing danger due to the use of social networks to promote the sale and recommendation of slimming products, diets, and distorted information regarding food (11).

In addition, numerous studies have observed an increase in the incidence of eating disorders (ED), which, although prevalent in both sexes, are more prominent in women and adolescents. ED include anorexia nervosa, bulimia nervosa, binge eating disorder, and other unspecified eating behaviors, which may range from episodes of compulsive eating to severe dietary restrictions, impacting individuals’ physical and mental health in various ways (12–14). The Global Burden of Disease Study from 2019 (GBD), recently published (2022), recognizes ED as mental illnesses and a public health problem in developed countries (15). In 2019, according to this study, 14 million adults suffered from ED of which nearly 3 million were children and adolescents. However, other authors argue that these data are underestimated, suggesting there are an additional 41.9 million cases. Santamuro D et al. state that their diagnostic focus was exclusively on anorexia nervosa and bulimia nervosa, which represent just the tip of the iceberg when it comes to ED (16).

In Spain, multiple studies have been conducted on the prevalence of ED within the population, and it is estimated that the diagnostic prevalence of ED is around 1–4%, with more than 3% for unspecified ED (17).

The development of ED involves many biological and psychosocial variables, but its relationship with the use of SM and the internet is a topic that many studies are increasingly focusing on (1), seeking to understand the influence these digital environments can have on ED (4).

In a recent study conducted by López-Gil et al., it is demonstrated that greater use of SM and higher levels of addiction to these platforms increase the likelihood of developing an ED. Specifically, the research highlights the use of Instagram, which has a stronger association with ED. Exposure to these images can lead to social comparison, increasing body dissatisfaction and fostering the development of ED (18).

Many analyses address the concept of body image, which is the image our brain synthesizes about our physical appearance, i.e., how we perceive ourselves (19, 20). Some resources on SM promote beauty ideals and cultures of “ideal thinness,” which can be detrimental to self-esteem and body image (4). In this way, some of these platforms promote an increase in body dissatisfaction, which is one of the most important factors in developing an ED, with a directly proportional relationship between the use of these platforms and the frequency of consuming such content (21–23).

Many researches support that many psychosocial and socio-cultural factors are involved in body image formation and, therefore, these factors may contribute to body dissatisfaction. These include the influence of advertising, the way children are brought up, parents’ beliefs about physical appearance and beauty references, peer relationships, etc. Children and adolescents access digital social environments in which thin beauty predominates at an early age. In addition, those who delay this contact with SM inevitably interact with peers who are absorbed in this digital culture of ideal thinness. So, in one way or another, they end up coming into contact with the aesthetic model of thinness and, consequently, they can develop preoccupations with weight and shape and dangerous attitudes that can lead to ED (24).

Furthermore, the diversity of blogs and pro-anorexia (pro-ana) and pro-bulimia (pro-mia) content that can be found through a few searches on Google or some SM has been analyzed before, with this information sometimes disguised as “losing weight easily,” “meeting people,” etc. These digital environments can serve as a refuge, support, or hiding place for people with ED, further reinforcing these harmful behaviors (21–23, 25–27).

It is important for health professionals to be fully aware of this reality, because ED can also lead to other complications such as menstrual disorders, reduced fertility, bone and cardiovascular pathology, among others. In addition, there are many comorbid mental health conditions such as depression, specific phobias, anxiety, obsessive compulsive disorder, etc., thus making ED one of the main causes of disability among young women (28–30).

This study aims to bring together all these concepts and interrelate them, primarily trying to qualitatively analyze different digital resources with pro-ana and pro-mia content found in the most prevalent SM today. This is of great interest to healthcare professionals to understand how this type of content affects patients with ED and how they manage to access it. Therefore, the objectives of this study are to qualitatively analyze pro-ana and pro-mia content on current dominant SM, understand the limitations and/or facilities users have for accessing this content, and investigate how consuming this content affects individuals who have developed ED or are susceptible to developing them.

## 2 Materials and methods

### 2.1 Study design

This study’s design follows current guidelines on qualitative research. Qualitative and quantitative methodologies are not exclusive; the goal of qualitative research is interpretation. Thus, values, experiences, and specific contexts become crucial, allowing for a more effective examination and deciphering of people’s behavior and situations under study. Qualitative research follows a circular research model in which the researcher must address the study comprehensively, enriching the understanding of the work (31). Through this methodology, the aim is to analyze the pro-ana and pro-mia content of the main audiovisual resources on the Internet, focusing on the most important SM today, to understand their relationship with ED.

### 2.2 Study sample, participants and context

**Figure 1:**
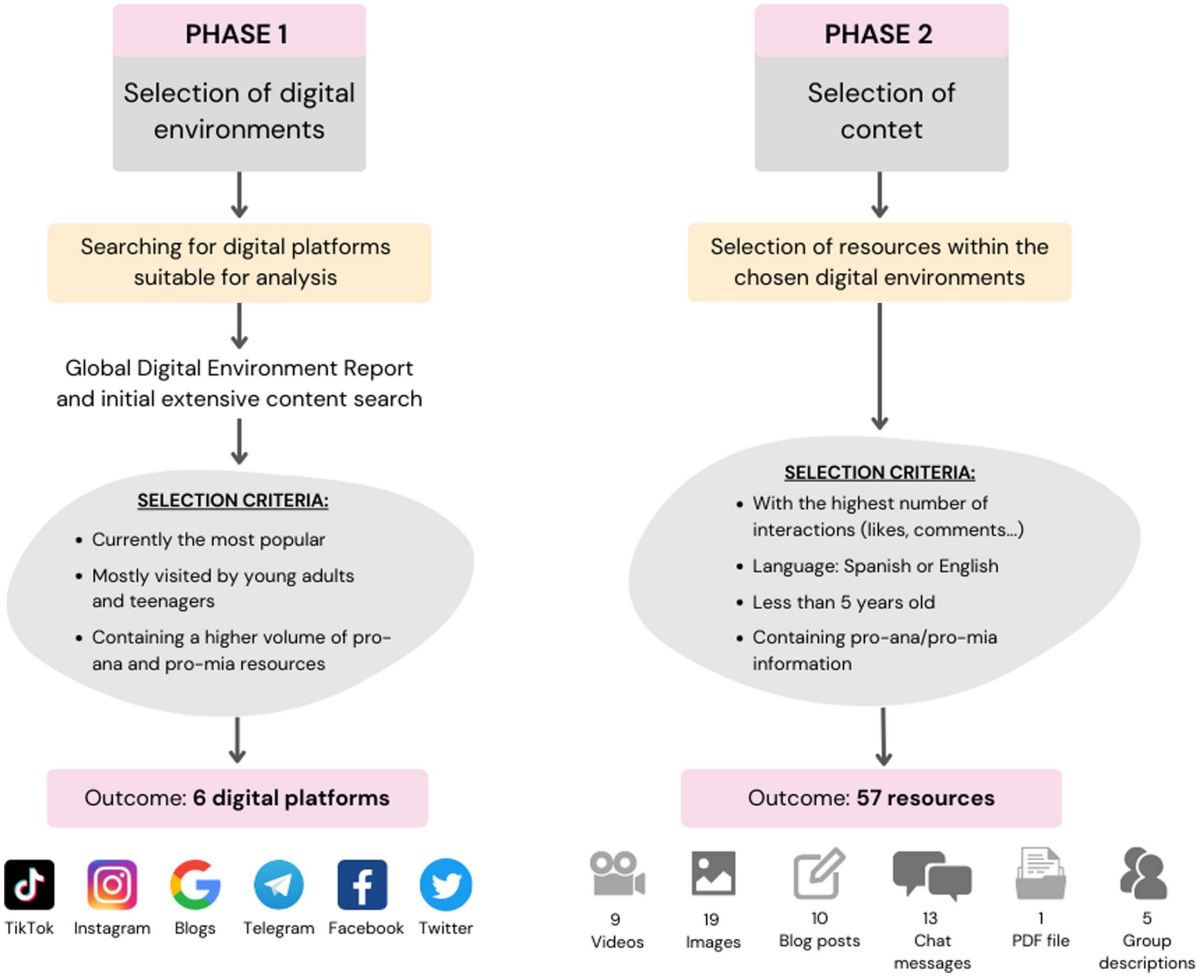
Study sample, instruments for data collection and fieldwork.

In qualitative research, there are no strict rules about the appropriate sample size for the study; instead, the gathered information guides the sampling. In this case, the process has been gradual, with non-random intentional sampling, studying videos, images, texts, blogs found through Google searches and chats on the following SM or the internet: TikTok, Instagram, Google blogs, Telegram, Facebook, and Twitter. Although resources from each SM were analyzed, a larger sample was extracted, especially from TikTok and Telegram, as they are the most used platforms by adolescents and young adults, according to the Global Report on the Digital Environment, compared to other SM. In addition, Telegram was further explored due to the wealth of enriching content and easy access. A total of 6 digital environments were studied (Figure 1).

### 2.3 Data collection instruments and fieldwork

**Figure 2:**
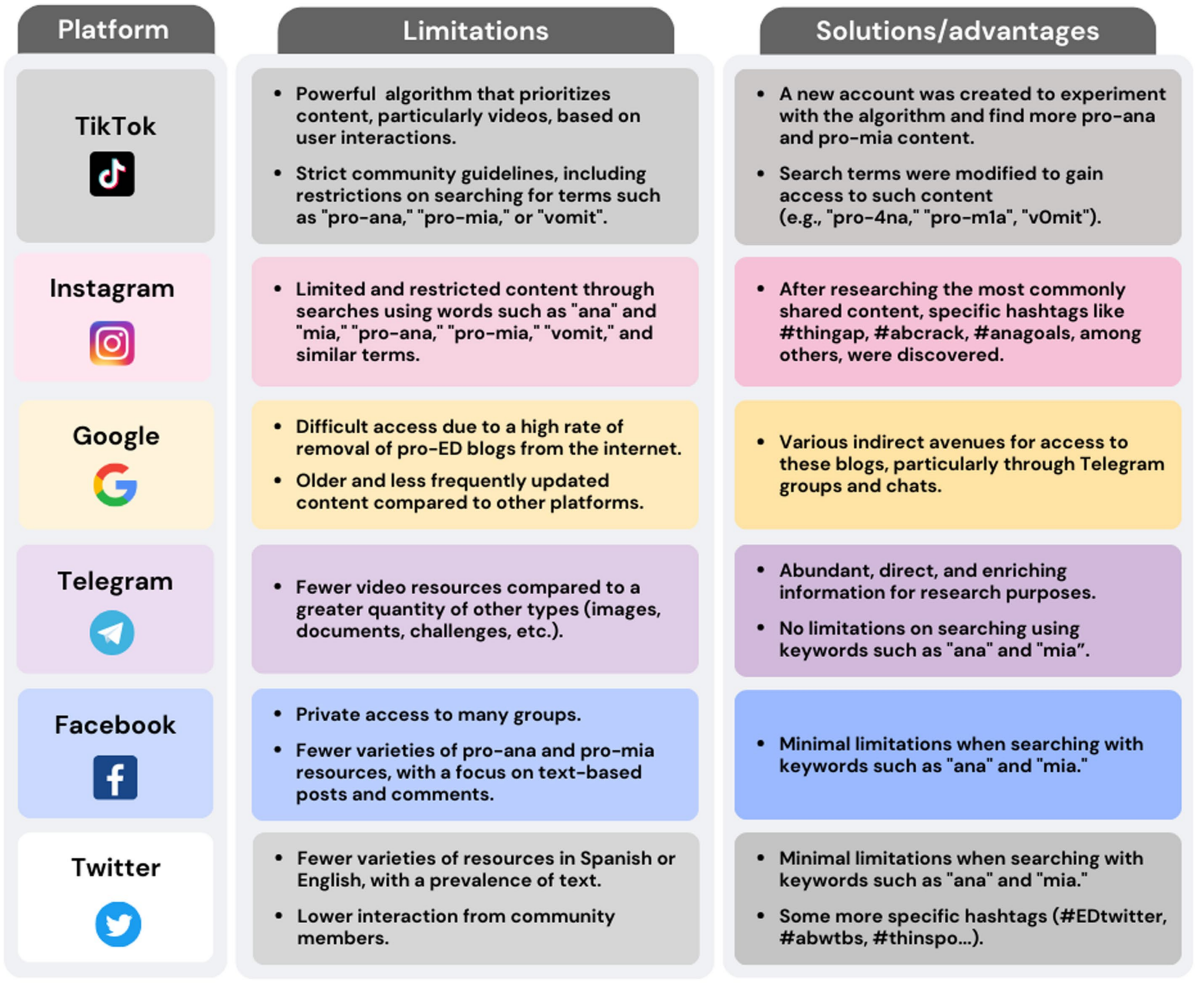
Limitations and solutions/advantages of each social network.

Fieldwork was conducted from September 2022 to January 2023. For information collection, 6 digital platforms were studied: TikTok, Instagram, Google blogs, Telegram, Facebook, and Twitter. Content that met the following criteria was selected: the most prominent content on each platform in terms of the number of interactions (likes, comments, group members, top positions, etc.), less than 5 years old, showing pro-ana and/or pro-mia content in English or Spanish. In addition to ‘ana,’ ‘mia,’ ‘pro-ana,’ and ‘pro-mia,’ the keywords used included: ‘anorexia’ ‘bulimia,’ ‘workout obsession,’ ‘weight loss tricks,’ ‘detox plans,’ ‘vomiting tips,’ ‘laxative use,’ ‘thinspo,’ and ‘body checking.’ This comprehensive list aimed to capture a broader spectrum of disordered eating behaviors and online content. Additionally, we used variations with numbers (e.g., ‘pr0-ana,’ ‘4na’) and specific hashtags like #bonespo, #edrecovery (misleading usage), #anafamily, #fastingchallenge, #thingap, #abcrack, #anagoals, #EDtwitter, #abwtts, and #thinspiration to bypass certain restrictions and filters applied by SM platforms. However, each platform presented its limitations, and the accessibility to this type of information varied from one digital environment to another, requiring the use of different keywords depending on the restrictions (Figure 2).

In total, 57 resources were analyzed: 9 videos, 19 images, 10 blog entries, 13 chat messages from Telegram groups, 1 PDF file, and 5 Facebook group descriptions, as well as the respective comments and interactions for each resource (Figure 1).

### 2.4 Data classification and analysis

All content was transcribed strictly and literally, including spelling mistakes. For the analysis, the number of members of each group, “likes,” retweets, comments, publication dates, and the textual transcription of the content, appearing in quotation marks, were specified. Each image and video were also detailed (including duration, tone used, accompanying music, and non-verbal language). English content was translated into Spanish for easier understanding.

**Figure 3:**
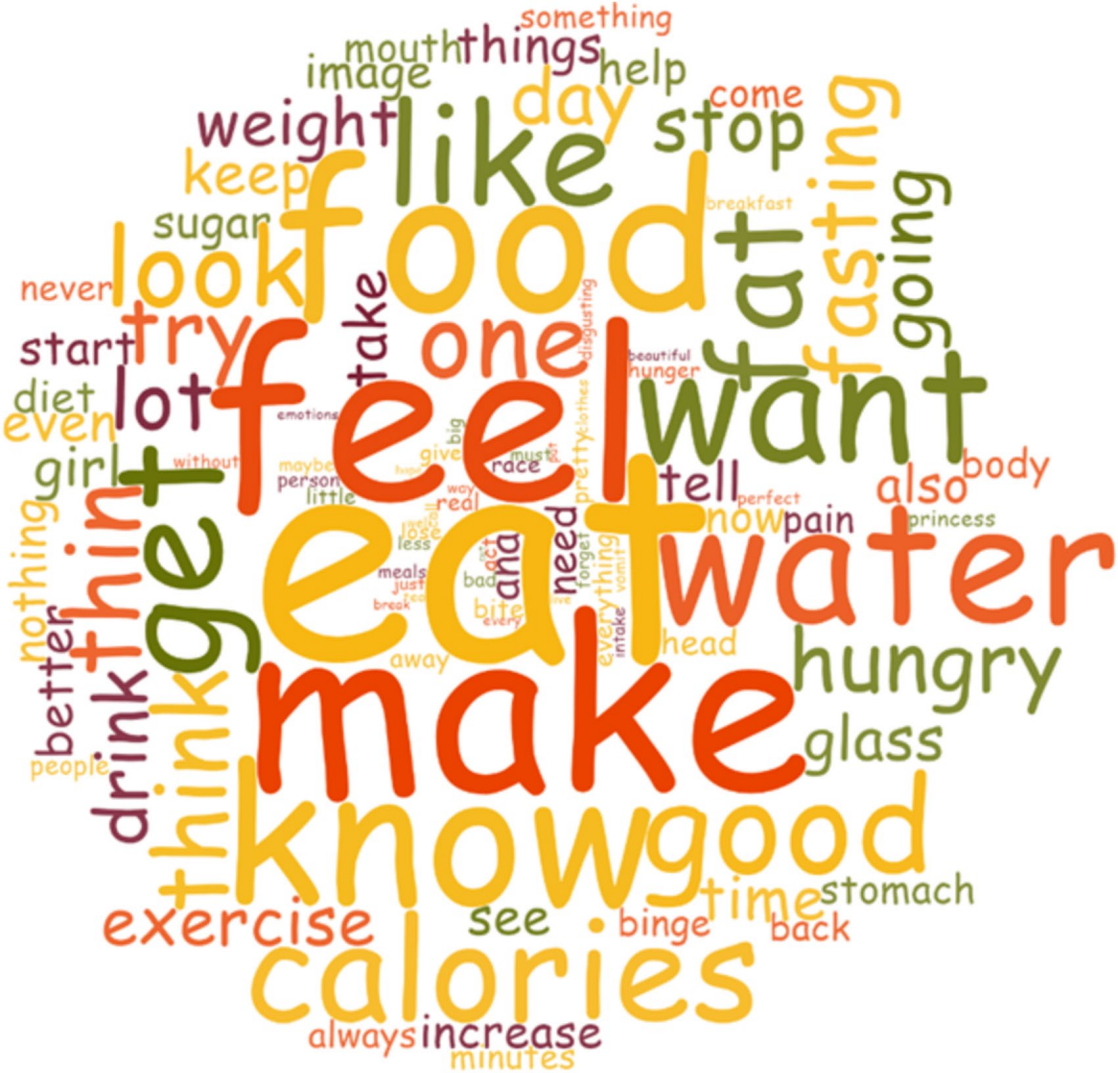
Word cloud from N-Vivo12 software.

To analyze and code the discourse, the data were entered into the N-Vivo12 software, a program for qualitative information analysis that allowed examining which words were repeated most frequently. After processing, a word cloud was extracted, where the size of each word’s letter is directly proportional to the frequency with which the word appeared. Additionally, the most repeated words are placed closer to the center of the cloud, while less repeated words are closer to the periphery (Figure 3). This tool has been used in previous studies and is considered a valid and reliable method for summarizing large amounts of information in a small space.

The discourse was analyzed from two perspectives: quantitative and qualitative. For the qualitative analysis, we took into account the frequency with which the content was repeated, the number of likes or dislikes each video received, the number of comments, the content’s positivity or negativity, and the kind of interactions that arose in the groups analyzed on the different platforms.

## 3 Results

From the word cloud analysis, it is concluded that the most repeated words are those related to food and body image, such as ‘eating’, ‘calories’, ‘thin’, ‘food’, ‘fasting’, ‘fat’, ‘hungry’, etc., with those words related to not eating/thinning being positively objectified and those related to eating/fattening being negatively objectified. Also, verbs such as ‘can’, ‘doing’, ‘want’, etc. stand out, accompanied by risky actions for ED.

The videos are generally about 10–20 s long on average, and show, like the images, thin women repeatedly. The videos are dominated by songs that talk about physical appearance, with an upbeat and fast-paced tone.

In order to synthesize the information, four categories were formed for greater comprehension, grouping those concepts and aspects that are most repeated in these digital communities.

**Figure 4:**
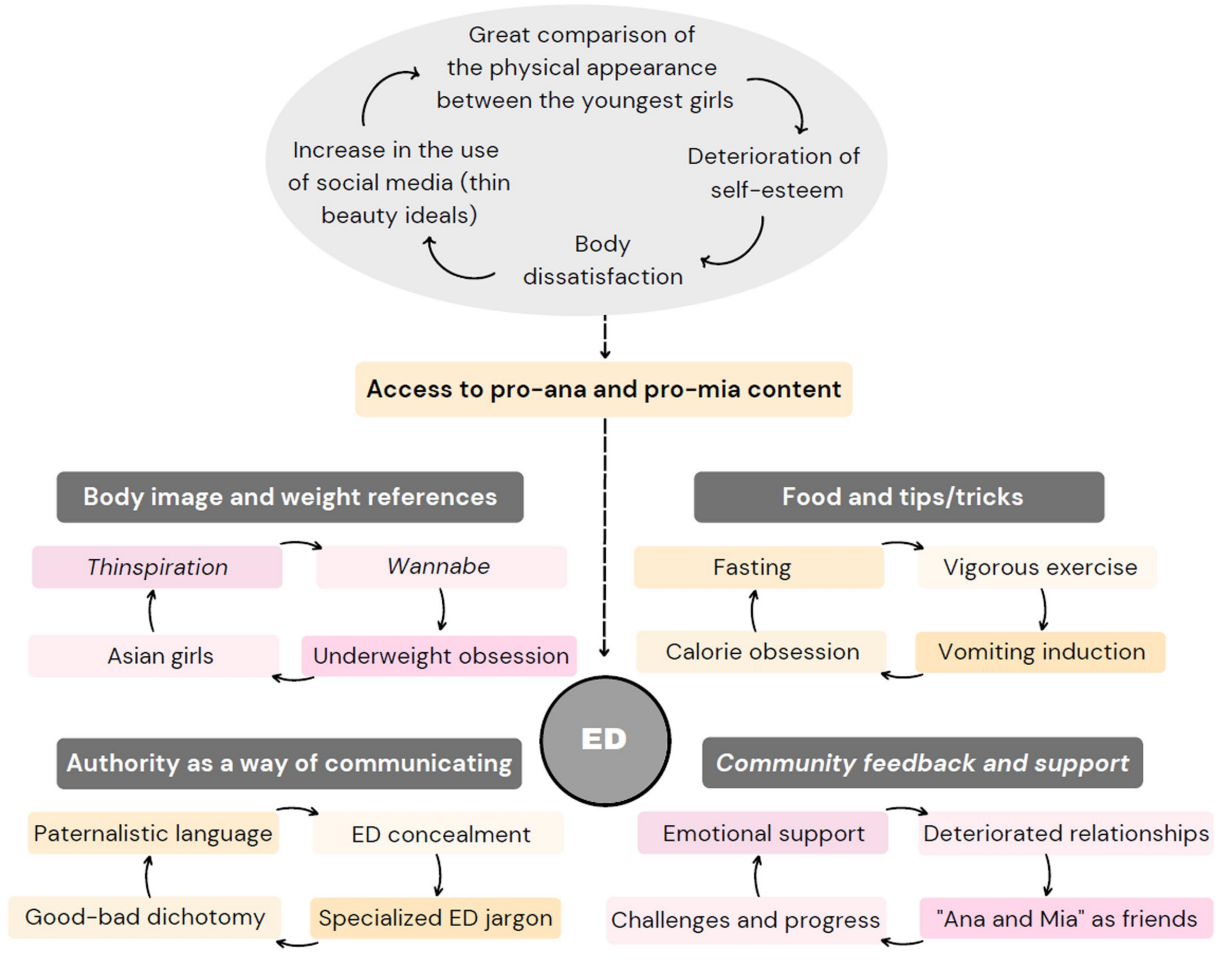
Factors involved in the development and/or aggravation of EDs.

The importance given to weight and body image stands out, for which an infinite number of tricks or tips are shared, promoting anorexic and bulimic behaviours, with specific slang predominating among the members, who share their progress perpetuating these risky behaviours and even promoting challenges. Therefore, the formation of the four categories has been done on the basis of these aspects (Figure 4), and at the end of each paragraph an example is written verbatim in inverted commas, including spelling mistakes.

### 3.1 Body image and weight references

The constant yearning for thinness stands out, with Asian girls as references in many cases, and a common desire is expressed for the vertical stripe on a flat and marked abdomen (known as abcrack), as well as for the thingap, which is the gap formed between the thighs when the legs come together in slim people. This is all part of the concept of thinspiration or thinspo, which means an inspiration in thinness, a term censored on many SM. Another closely related term is wannabe, which refers to people who do not have an ED but would like to, in order to be thin. In addition, bones marked on the skin are directly related to thinness, perfection and femininity. The ‘disadvantages of being fat versus being thin’ are often stated, and it is common to find many before-and-after images of physical change, as well as images or accounts of self-harming attempts.

*“(…) Because to be successful you need to be thin. Because no man would rather have sex with a fat girl than a thin girl. Because all the fat girls envy you. Because if you are thin, you are everything.”*

*(Anonymous, from a blog found in Google).*

### 3.2 Food and tips/tricks

The importance of calories is highlighted and a variety of applications that allow users to count calories and read food labels are recommended, which can be significantly detrimental to people suffering from ED. Thus, fasting is one of the ‘top tips’ in this community, along with intense exercise or purging by vomiting (vomiting with finger is relegated to second place, and tricks such as brushing the tongue, spinning around a lot to get dizzy or drinking water with salt are practised). In addition, satiating substitutes that provide hardly any calories, such as tea, water, chewing gum, laxatives, sweeteners, apples or ice, are popular. Following the same logic, there is great concern about the number of kilograms on the scale, with hundreds of comments on weight, with a predominance of comparisons. The conclusion is that food and calorie intake are perceived as unpleasant, while there is a strong obsession with vomiting, fasting, low weight and intense physical exercise.

*“WATER DIET. IT’S VERY SIMPLE, you just have to drink water. Breakfast: two glasses of water. Mid-morning: one glass of water. Lunch: three glasses of water and an apple. Afternoon snack: two glasses of water or a red tea. Dinner: two glasses of water.”*

*(Anonymous, from a chat in a group of Telegram).*

### 3.3 Authority as a form of communication

Paternalistic communication predominates in this environment, in which there is one person who, in an authoritarian manner, tries to educate the other to learn and follow the ‘right’ steps to be thin. It is not a communication between equals, but there is one person who leads and another who obeys, with frequent use of imperative speech, derogatory words (the most used is “Fat!”) and guidelines ordered even in the format of commandments, using messages with a hypnotizing tone that try to instill guilt in the victim through a vocabulary full of harshness. Moreover, they emphasize a “bad-good dichotomy” (providing an additional nuance of radicalism and indoctrination) and the tricks for hiding the ED: from dirtying the dishes without eating to lying to the doctor or wearing baggy clothes.

*“HUNGER IS NOT YOUR ENEMY, YOU WILL FEEL WONDERFUL EVERY TIME YOU ARE HUNGRY; WATCH OTHER PEOPLE EATING. YOU ARE SUPERIOR TO THEM BECAUSE YOU DON’T NEED TO EAT. THEY EAT AND THEY GET FATTER AND FATTER AND FATTER AND FATTER. YOU DON’T WANT TO LOOK LIKE THAT, YOU DON’T WANT TO BE LIKE THEM.”*

*(Anonymous, from a chat in a group of Telegram).*

### 3.4 Community feedback and support

Users post their daily lives and their progress towards slimness in these communities as if it were a diary. They support each other to “stay strong” and share a variety of resources to help them achieve weight loss (weekly fasting or low-calorie challenges, calorie counting apps, PDF files with nutritional tables.). These people (mostly girls) call themselves princesses, as they do with “Ana and Mia,” which is their way of calling anorexia and bulimia, respectively, as if they were their friends. Comments supporting these behaviours abound, with praise for thinness and constant interpersonal comparison in terms of weight and body image. Often, they report not feeling understood in their real environment, which may explain the support of this community, full of people in the same vulnerable situation.

*“I did another 30hs. I broke it up with baked aubergine and a bit of potato … I HAD A HORRIBLE TIME. I HAVE: Diarrhoea, Vomiting, Belly ache, Headache, I smell food and it makes me sick, I had 2 fainting spells (…)”*

*(Anonymous, from a chat in a group of Telegram)*

## 4 Discussion

This study aimed to qualitatively analyze pro-ana and pro-mia content on different digital platforms to understand its potential impact on individuals with ED or those at risk. The results revealed a consistent pattern across the analyzed platforms, with content promoting extreme thinness, providing tips on weight loss, and fostering a positive sentiment towards low body weight. Users engaged in these digital environments expressed admiration for thin bodies, sought advice on achieving similar results, and shared their own experiences with ED. These findings are consistent with previous work, which also highlights the relationship of problematic use of the Internet and SM with the development or aggravation of EDs, with anorexia and bulimia predominating and being more frequent in females and adolescents (32–34). In fact, EDs have become the third leading cause of chronic disease in adolescent girls in industrialized countries (13).

This may be one of the reasons why almost all participants or creators of this content are young women. In addition, most of the audiovisual content found in this type of community shows slim women, which further supports this point. Similarly, in general, women tend to bear a higher social pressure regarding physical appearance, which is even more accentuated at adolescence, where comparison between people is rather common. Consequently, frequenting SM for the purpose of viewing and interacting with images of other people is a more common practice among women, while men tend to access these digital environments for other purposes, such as interacting with friends (35–37). Thus, adolescents and young adults (particularly women) often find themselves in a constant self-evaluation of their physical appearance through comparison on SM with users who meet the socio-cultural ideals of slim beauty of the current era. On many occasions, the number of “likes” is equated with beauty status or self-esteem, even becoming a measure of personal merit on SM (38–40).

In young adults, exposure to food-related content in SM like TikTok videos has been found to have positive direct effects on body appreciation and diet intentions, while also having a negative direct effect on body dissatisfaction. This suggests that viewing content related to healthy eating and diet practices can enhance how young adults feel about their bodies and motivate them to pursue healthier eating habits. However, it also underscores a potential downside, as such content can simultaneously exacerbate feelings of dissatisfaction with their bodies. Furthermore, positive mood plays a crucial role in this dynamic, as it mediates the relationship between social comparison and diet intentions. When young adults compare themselves to others in a positive light, it can enhance their mood, which in turn, influences their intentions to follow certain dietary practices. This highlights the complex interplay between emotional states and the impact of SM content on personal health behaviors (41).

Insecure attachment is recognized as a general risk factor for ED, with emotion dysregulation proposed as a potential mechanism through which attachment insecurity influences eating disorder psychopathology. Individuals with insecure attachment styles, characterized by anxiety and avoidance, often struggle with regulating their emotions effectively. This emotional dysregulation can lead to maladaptive eating behaviors as a way to cope with their emotional distress (42). These findings are consistent with our study, where participants seek community feedback and support through the use of SM. SM platforms provide a space for individuals to share their experiences and receive feedback, which can be both beneficial and detrimental. On one hand, it offers a sense of community and support for those looking to improve their eating habits and body image. On the other hand, it can also perpetuate harmful comparisons and reinforce negative self-perceptions (43).

The analysis of specific platforms provided insights into the nature of pro-ana and pro-mia content on each platform. On TikTok, users shared their daily routines centered around low-calorie diets and exercise, garnering admiration from followers. Instagram content featured images of thin bodies with captions promoting extreme dieting and exercise, eliciting a mix of admiration and concern from users. Google Blogs contained personal experiences with ED and advice on hiding the disorders, fostering a positive tone towards thinness. Telegram messages shared tips on fasting and calorie restriction, with group members providing support for weight loss goals. Facebook group descriptions emphasized support for individuals with ED while promoting weight loss. Twitter content expressed a desire for thinness, accompanied by tips on achieving weight loss and maintaining a low body weight, as well as images of physical changes.

The regular visit of SM by younger adults encourages more of this digital comparison, which can negatively affect self-esteem, with feelings of body dissatisfaction, thus creating a vicious cycle between these elements. Girls not only tend to show a greater concern for appearance, but also present greater mental health problems associated with the use of SM, particularly depressive symptoms and the development of ED (44). On the other hand, in EDs there is often a distorted body image (body dysmorphia), which can generally lead to visiting digital content that motivates weight loss and the practice of health-risk behaviours (45–47).

A large number of pro-ana and pro-mia communities were observed on the Internet and on SM, with many tips and tricks promoting dangerous habits: methods for vomiting, extreme diets bordering on complete fasting, intense exercise challenges, images of people with marked thinness. Participants share their progress and thus establish strong social bonds between people in the same situation of weakness through exchanging photos, experiences, advice and feelings, creating a kind of emotional community in which members find social support, which further encourages the support of these behaviors (48, 49). Moreover, most users experience deteriorated social relations in their real world, so they try to hide their condition from their loved ones. Therefore, these digital environments are perceived by these individuals as forms of help and emotional release where they feel identified with others. In addition, that specific jargon and paternalistic/authoritarian mode of communication used, further consolidates this feeling of belonging to a group.

Furthermore, during the confinement due to the COVID-19 pandemic, people had to experience a forced social distancing, which encouraged an increase in the use of SM, as well as an increase in the range of accounts to follow. Self-esteem was deteriorated in a part of the population, and health problems increased (ED, depression, anxiety). This was related to a rise in the use of SM, the degree of body dissatisfaction and the desire for thinness on the part of many of the users, thus forming a vicious circle (21). Human beings are social by nature, so, by not experiencing interpersonal relationships in the real or physical world, an increase of FOMO was observed (fear of missing out: a worried feeling that you may miss exciting or important social events, many of them shared on SM) (50, 51). This may explain, as a further factor, the increased use of SM during confinement and the need to belong to a group in pro-ana and pro-mia communities.

On the other hand, the accelerated growth in e-commerce could be attributed to people transitioning from traditional physical store visits to online shopping. This shift in purchasing behavior may significantly impact food choice motives, as individuals can access a broader range of food options online and make choices based on convenience, availability, and health considerations. Consequently, online shopping can alter dietary habits, potentially encouraging unhealthy eating patterns. The ease of access to various food products, including those that are less nutritious, may lead to impulsive or convenience-driven choices. Furthermore, excessive SM engagement can compromise the ideal body image, thus increasing the likelihood of engaging in disordered eating behavior (52, 53).

Healthcare professionals and educators play a crucial role in addressing this issue by promoting digital literacy and awareness among young individuals. It is essential to educate individuals about the potential risks of engaging with pro-ana and pro-mia content, emphasizing the importance of a healthy body image and the dangers of extreme dieting practices. Additionally, efforts should be made to monitor and regulate the dissemination of such content on SM platforms, implementing measures to restrict its availability and protect vulnerable individuals.

### 4.1 Limitations and strengths of the study

This study has some limitations that should be considered. The non-random intentional sampling method may introduce selection bias, as the chosen platforms and content may not be representative of the entire spectrum of pro-ana and pro-mia content on the internet. Additionally, the analysis was limited to content in English and Spanish, which restricts the generalizability of the findings, as online content in other languages and cultural contexts was not included. The qualitative nature of the analysis provides in-depth insights into the content but may not capture the full extent of the issue. Furthermore, each SM platform presented specific limitations like search restrictions, content moderation algorithms and issues related to accessibility. These factors should be taken into account when interpreting the results and considering their applicability in broader contexts.

On the other hand, qualitative studies evolve as they progress; researchers do not operate by design, but rather, they design as they act. The scientific objective of this methodology is not to establish causal relationships to explain phenomena through universal laws, but to understand the nature of the phenomenon under study. These types of research methods make patient experiences accessible, and the information they provide is closely related to the experience of professionals. Therefore, the qualitative nature of the analysis offers detailed information about the content, although it may not capture the entirety of the problem.

Each social network has strict community rules that make it possible to regulate the content that is shared, which has been an important limitation for the progress of the study, as many of the resources found were being eliminated when infringements of the rules were detected. In addition to the regulations attached to SM, there is also an indirect restriction of information because, although there is no specific legislation in Spain that controls this type of online content, there are some laws and regulations that indirectly limit its exposure.

The findings of this study contribute to the growing body of literature on the relationship between problematic internet use and ED, and future research could employ a more extensive and diverse sample to enhance the generalizability of findings and explore the cultural variations in pro-ana and pro-mia content.

## 5 Conclusion

Problematic internet use, particularly the consumption of pro-ana and pro-mia content on SM platforms, is associated with the development and exacerbation of ED. This qualitative analysis of content on TikTok, Instagram, Google Blogs, Telegram, Facebook, and Twitter revealed consistent themes of promoting extreme thinness, providing tips on weight loss, and fostering a positive sentiment towards low body weight. Users engaged in these digital environments (especially young women) expressed admiration for thin bodies, sought advice on achieving similar results, and shared their own experiences with ED. Furthermore, COVID-19 pandemic increased the use of SM, aggravating body dissatisfaction and promoting feelings of FOMO, which constitutes a combination of factors that feed off each other in a positive way.

Healthcare professionals, educators, and digital platforms should collaborate to address the dissemination of pro-ana and pro-mia content, implement preventive measures, and promote digital literacy and awareness among adolescents and young adults. By understanding the nature of such content and its potential impact, it is possible to develop targeted interventions to mitigate the negative consequences and protect vulnerable individuals from the harmful effects of problematic internet use.

## Data Availability

The raw data supporting the conclusions of this article will be made available by the authors, without undue reservation.
